# Patterns and prevalence of ophthalmic self-medication in Bulgaria: results from a cross-sectional survey

**DOI:** 10.3389/fmed.2026.1830788

**Published:** 2026-05-29

**Authors:** Mladena Radeva, Elitsa Hristova, Andreas Kontny, Zornitsa Zlatarova, Igor Resnick

**Affiliations:** Medical University of Varna, Varna, Bulgaria

**Keywords:** Bulgaria, online survey, ophthalmology, self-medication, self-treatment

## Abstract

**Background:**

Ophthalmic self-medication, defined as the use of over-the-counter eye medications without prescription, represents a significant public health concern due to potential complications such as delayed diagnosis, antimicrobial resistance, and vision impairment. Despite its global prevalence, data on this practice in Bulgaria remain limited. This study aimed to comprehensively assess the prevalence, patterns, determinants, and self-reported outcomes of ophthalmic self-medication among adults in Bulgaria.

**Methods:**

A cross-sectional online survey was conducted between March and May 2025 among adults aged ≥18 years residing in Bulgaria. An anonymous questionnaire comprising 15 items assessed sociodemographic characteristics, ocular complaints, motivations for self-medication, perceived safety and knowledge, and self-reported outcomes. A total of 1,000 respondents were included in the analysis. Descriptive statistics and inferential analyses (Pearson’s chi-square and Fisher’s exact test with Monte Carlo simulation) were performed, with statistical significance set at *p* < 0.05.

**Results:**

The sample was predominantly female (90.8%), aged 31–50 years (75.6%), highly educated (85.1%), and employed (83.1%). The prevalence of ophthalmic self-medication was 63.7%. The most common complaints were infectious conditions (37.3%) and dry-eye symptoms (23.1%). Primary motivations included lack of time to seek specialist care (41.1%) and the perception that consultation was unnecessary (33.7%). Most participants (66.2%) considered self-medication safe, while 39.6% reported poor or no knowledge of ophthalmic medications. Among self-treated respondents (*n* = 637), adverse reactions were rare (0.6%), and 34.5% sought ophthalmological consultation, mainly for routine examination (20.6%). Significant associations were observed between age and self-medication (*p* < 0.001), type of complaint and motivation (*p* < 0.001), and adverse reactions and consultation (*p* < 0.001), whereas no significant associations were found with gender, education, or perceived safety.

**Conclusion:**

Ophthalmic self-medication was highly prevalent in this Bulgarian sample and was primarily driven by accessibility barriers and perceived low necessity for specialist consultation. Although adverse events were rarely reported, the discrepancy between perceived safety and self-assessed knowledge highlights potential risks. These findings underscore the need for targeted patient education, improved access to ophthalmic care, and pharmacist-supported referral strategies to promote safer self-care practices.

## Introduction

Self-medication is defined as the use of medicinal products by individuals to treat self-recognized symptoms or disorders, or the continued use of previously prescribed medications for recurrent conditions. In ophthalmology, this practice commonly involves over-the-counter eye drops, ointments, or gels used for symptoms such as redness, dryness, itching, or ocular discomfort. These symptoms may reflect a wide spectrum of underlying conditions, ranging from minor irritations to more serious diseases, including infections, allergic disorders, or glaucoma.

Ophthalmic self-medication represents a significant global public health concern, as inappropriate use of ocular medications may lead to adverse outcomes such as delayed diagnosis, progression of underlying disease, antibiotic resistance, corneal toxicity, or even permanent vision impairment. Reported prevalence rates vary widely across populations, reaching up to 73.6% in certain settings, particularly in regions with limited access to specialized care and easy availability of non-prescription medications. Previous studies have identified key contributing factors, including limited awareness, economic constraints, and the widespread availability of over-the-counter products (1–4).

In European contexts, including Bulgaria, self-medication practices are often facilitated by community pharmacies, where individuals frequently seek advice influenced by advertising, online resources, and informal recommendations from peers or family members. While pharmacist involvement may support appropriate use in minor conditions, the accessibility of ophthalmic medications through non-prescription channels may also contribute to misuse and delayed professional evaluation.

Despite the relevance of this issue, data on ophthalmic self-medication in Bulgaria remain scarce. Although the healthcare system provides access to specialized ophthalmic services, barriers such as limited availability of appointments, geographic disparities, and time constraints may influence healthcare-seeking behavior. Existing local evidence suggests that patients frequently resort to over-the-counter medications for minor ocular complaints, often to save time or due to difficulties in accessing medical consultation (5). This highlights the need for further investigation into the patterns, determinants, and outcomes of ophthalmic self-medication within the Bulgarian population (6, 7).

The potential risks associated with self-medication are further compounded by gaps in patient knowledge and variability in perceived safety. Inappropriate use of antibiotic or corticosteroid eye drops, for example, may result in resistance, toxicity, or worsening of undiagnosed conditions. Differences between perceived safety and actual clinical risk emphasize the importance of generating context-specific evidence to inform public health strategies and clinical practice (8, 9).

This study aimed to comprehensively assess the prevalence, patterns, determinants, and self-reported outcomes of ophthalmic self-medication among adults in Bulgaria, addressing the existing evidence gap and providing context-specific data relevant to public health and clinical practice.

## Methods

### Study design and study period

This study employed a nationwide cross-sectional survey design to investigate the prevalence, practices, motivations, perceptions, and self-reported outcomes of self-medication with ophthalmic medications among the Bulgarian population. The survey was conducted over a three-month period in 2025, from early March to late May.

The study employed a convenience sampling approach, as participants were recruited voluntarily through online platforms. As a result, the sample reflects a digitally active and self-selected population rather than a probability-based representation of the general Bulgarian population.

### Study setting and recruitment

Data were collected using an anonymous online questionnaire distributed exclusively through social media channels, including Facebook, Instagram, and LinkedIn. The survey was shared through public groups, reposted across user networks, and promoted through targeted online dissemination in order to reach participants from different demographic backgrounds and geographic areas of Bulgaria. No financial or material incentives were offered for participation.

This recruitment strategy enabled rapid and broad dissemination of the questionnaire but also reflects a convenience sampling approach based on voluntary participation among digitally active populations.

### Participants and eligibility criteria

Participation was voluntary and anonymous. Individuals were eligible to participate if they were aged 18 years or older, resided in Bulgaria, and provided electronic informed consent before accessing the questionnaire. Responses from participants younger than 18 years, submissions without consent, and clearly incomplete questionnaires lacking essential demographic or outcome information were considered ineligible for analysis.

### Survey administration and prevention of duplicate responses

The questionnaire was hosted on a digital survey platform designed to ensure accessibility and ease of completion across desktop and mobile devices. To reduce the risk of repeated participation, the survey settings were configured to allow only one submission per IP address. No personally identifiable information was collected, and all responses were analyzed in anonymized form.

### Sample size

No formal *a priori* sample size calculation was performed, as the study was designed as an exploratory observational survey based on voluntary participation. All eligible completed responses collected during the study period were included in the final dataset, resulting in a total sample of 1,000 participants.

### Questionnaire development

The questionnaire was developed by the authors based on a review of the relevant literature on self-medication practices, ophthalmic health behaviors, and previously published survey-based studies in ophthalmology and public health. The instrument was originally designed in Bulgarian, the native language of the target population; therefore, forward–backward translation procedures were not applicable. Prior to dissemination, the questionnaire underwent internal review and refinement by the research team to improve clarity, relevance, and comprehensibility.

### Questionnaire content and structure

The final questionnaire consisted of 15 items, including two conditional questions triggered by prior responses. Most items were closed-ended with predefined response options, and multiple selections were permitted where appropriate. Selected questions also included optional open-text fields allowing participants to provide additional details.

The questionnaire was organized into the following thematic domains:

- Sociodemographic characteristics – age, sex, education level, and employment status.
- Self-medication behavior – use of ophthalmic medications without prescription.
- Clinical context – ocular complaints or symptoms motivating self-medication.
- Motivations for self-medication – reasons for avoiding, delaying, or not seeking professional consultation.
- Perceptions and knowledge – perceived safety of self-medication and self-assessed knowledge regarding ophthalmic medications.
- Self-reported outcomes – adverse reactions, worsening of symptoms, and consultation with an ophthalmologist after self-medication.

### Outcome measures

The primary outcome was the prevalence of ophthalmic self-medication, defined as self-reported use of eye medications without a physician’s prescription. Secondary outcomes included motivations for self-medication, types of ocular complaints, perceived safety, self-assessed knowledge, reported adverse reactions, worsening of symptoms, and healthcare-seeking behavior following self-medication.

### Statistical analysis

Statistical analyses were performed using IBM SPSS Statistics version 27.0 (IBM Corp., Armonk, NY, United States). Descriptive statistics were used to summarize participant characteristics and questionnaire responses. Categorical variables were presented as frequencies and percentages.

Associations between categorical variables were examined using Pearson’s chi-square test of independence when expected cell counts met standard assumptions (expected frequencies ≥5 in at least 80% of cells). Degrees of freedom were calculated as (rows − 1) × (columns − 1). For contingency tables containing multiple categories or low expected frequencies, Fisher’s exact test with Monte Carlo simulation was applied, as appropriate. Statistical significance was defined as a two-sided *p*-value < 0.05.

Because some participants did not complete all questionnaire items, the sample size varied slightly across specific analyses. Missing data were minimal and were handled using listwise deletion for inferential analyses. No adjustments for multiple comparisons were applied, as the analyses were exploratory in nature.

### Ethical considerations

This study involved human participants who voluntarily completed an anonymous online questionnaire. Electronic informed consent was obtained from all participants prior to participation. The study was conducted in accordance with the Declaration of Helsinki and approved by the institutional ethics committee (approval number: #1A/26.02.2025).

## Results

The study included a total of 1,000 participants and provides an overview of demographic characteristics, ocular complaints, motivations for self-medication, perceptions of safety and knowledge, prevalence of ophthalmic self-medication, and associated outcomes.

### Participant characteristics

Of the 1,000 respondents, 908 (90.8%) were women and 92 (9.2%) were men. The most represented age groups were 31–40 years (38.3%) and 41–50 years (37.3%), followed by 51–60 years (10.5%) and 61–70 years (10.2%). Individuals older than 70 years accounted for 2.6% of the sample.

Most participants had higher education (85.1%), while 10.3% reported secondary education and 3.8% secondary-specialized education. Regarding employment status, 83.1% were employed, 7.4% were retirees, and the remainder were students, unemployed, or on maternity leave. Detailed demographic data are presented in Table 1.

**Table 1:** Demographic characteristics of participants.

Variable	Category	*n*	%
Gender	Female	908	90.8
	Male	92	9.2
Age (years)	18–30	11	1.1
	31–40	383	38.3
	41–50	373	37.3
	51–60	105	10.5
	61–70	102	10.2
	>70	26	2.6
Education	Secondary	103	10.3
	Secondary–specialized	38	3.8
	Higher	851	85.1
Employment status	Employed	831	83.1
	Retired	74	7.4
	Unemployed	50	5.0
	Other (Student/Maternity)	20	2.0
	Missing/No response	25	2.5

### Ocular complaints and motivations for self-medication

The most frequently reported ocular complaints motivating self-medication were infectious conditions such as conjunctivitis (37.3%), followed by dry-eye symptoms (23.1%), redness without additional symptoms (15.1%), allergic manifestations such as itching or burning (8.2%), and foreign-body sensation (4.1%). Other minor complaints accounted for the remaining responses.

The main reasons for using ophthalmic medications without prescription were lack of time to visit a specialist (41.1%), considering consultation unnecessary (33.7%), and inability to schedule an appointment with an ophthalmologist (9.4%). Less frequent reasons included trust in pharmacist recommendations (5.4%), seeking advice online (3.0%), and other personal or financial circumstances. These findings are summarized in Table 2.

**Table 2:** Ocular complaints and motivations for self-medication.

Ocular complaint	*n*	%	Primary motivation	*n*	%
Infectious conditions (conjunctivitis)	373	37.3	Lack of time to visit specialist	411	41.1
Dry-eye symptoms	231	23.1	Considered visit unnecessary	337	33.7
Allergic itching/burning	82	8.2	Unable to obtain appointment	94	9.4
Redness without secretion	151	15.1	Trust in pharmacist	54	5.4
Foreign-body sensation	41	4.1	Internet advice	30	3.0
Other minor complaints	122	12.2	Other/financial/abroad	14	1.4

### Types of ophthalmic preparations used

Among respondents who reported self-medication, the most commonly used ophthalmic preparations were antibiotic eye drops, followed by artificial tears and combined antibiotic/corticosteroid preparations. Vasoconstrictor drops used for ocular redness were also frequently reported. Less commonly used products included anti-allergic eye drops, non-steroidal anti-inflammatory eye drops, and pure corticosteroid preparations. Use of glaucoma medications without prescription was rare.

### Perceptions of safety and self-assessed knowledge

Regarding perceptions of safety, 66.2% of respondents considered self-medication safe, 25.3% were unsure, and 8.5% considered it risky.

**Figure 1:**
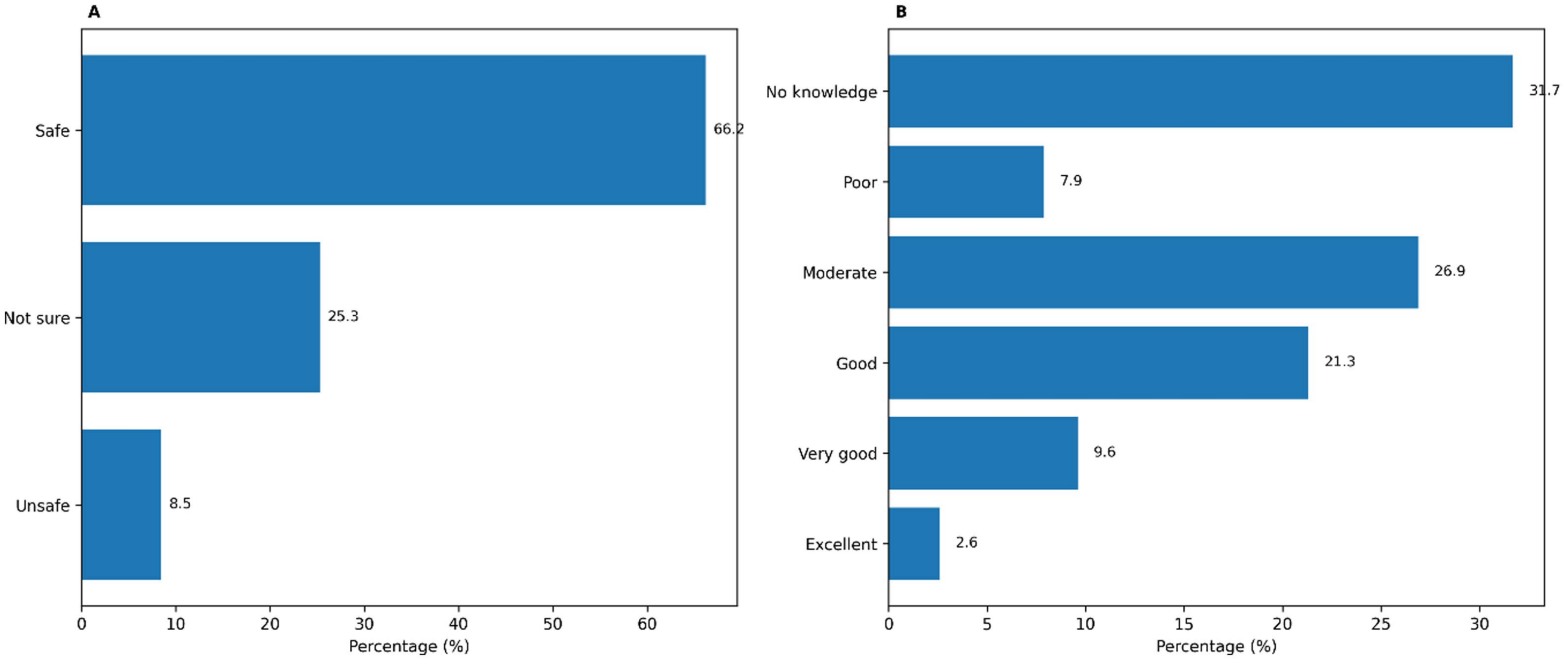
Perceived safety and self-assessed knowledge regarding ophthalmic self-medication. Panel **A** presents participants’ perceptions of the safety of self-medication with ophthalmic preparations. Panel **B** summarizes respondents’ self-assessed level of knowledge regarding ophthalmic medications. Values are presented as percentages of respondents.

Self-assessed knowledge regarding ophthalmic medications varied across respondents. Overall, 31.7% reported no knowledge, 7.9% poor knowledge, and 26.9% moderate knowledge. In contrast, 21.3% rated their knowledge as good, 9.6% as very good, and 2.6% as excellent. These distributions are illustrated in Figure 1.

### Prevalence and outcomes

The overall prevalence of ophthalmic self-medication was 63.7% (*n* = 637), whereas 36.3% of participants reported no use of ophthalmic medications without prescription.

Adverse reactions were rarely reported (*n* = 4), corresponding to 0.4% of the total study population and 0.6% when calculated among participants who engaged in self-treatment.

**Figure 2:**
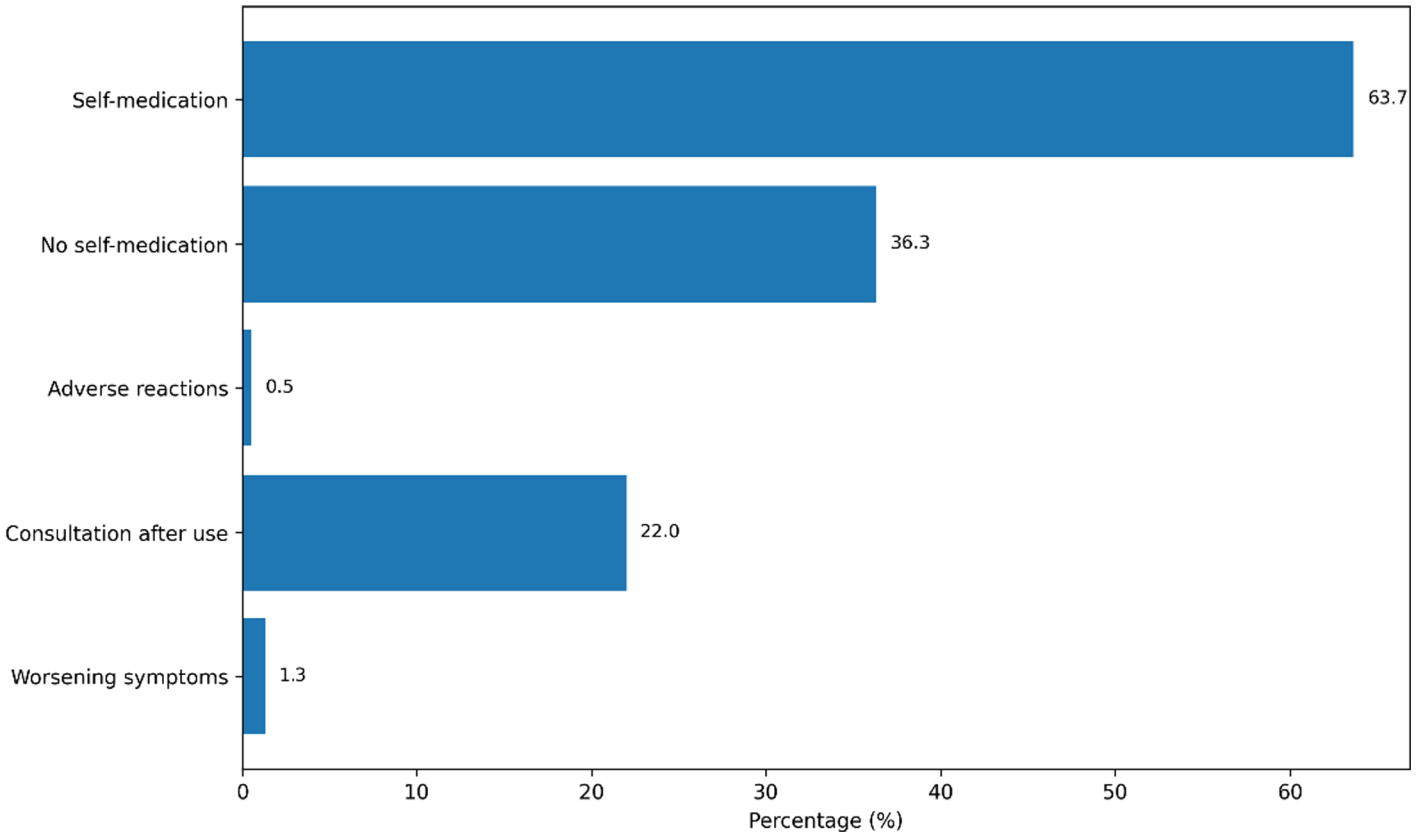
Prevalence and self-reported outcomes of ophthalmic self-medication. The figure illustrates the proportion of respondents reporting self-medication, adverse reactions, consultation with an ophthalmologist after self-medication, and worsening of symptoms. Values are presented as percentages.

Following self-medication, 220 participants sought ophthalmological consultation, corresponding to 22.0% of the total sample and 34.5% of self-treated respondents. Most consultations were for routine examination (*n* = 206), while 13 participants (1.3% of the total sample; 2.0% of self-treated respondents) reported consultation due to worsening of symptoms. These findings are presented in Figure 2.

The survey did not assess treatment effectiveness, placebo effects, or objective clinical outcomes. Therefore, no conclusions can be drawn regarding the adequacy or effectiveness of specific treatments based on these self-reported data.

### Associations between variables

Associations between self-medication and selected demographic variables were examined using contingency table analyses. A statistically significant association was observed between age group and self-medication status (χ^2^(5) = 38.7, *p* < 0.001). In contrast, no significant association was found between self-medication and gender (χ^2^(1) = 2.99, *p* = 0.084).

When education was regrouped into two categories (secondary/secondary-specialized vs. higher education), the association with self-medication remained non-significant (χ^2^(1) = 2.47, *p* = 0.116).

The relationship between perceived safety of self-medication and reported adverse reactions was also examined and found to be non-significant (χ^2^(2) = 0.489, *p* = 0.783). Despite the very low number of reported adverse reactions, this analysis was conducted to assess whether perceived safety corresponded to the occurrence of adverse reactions.

A significant association was identified between the type of ocular complaint and the primary motivation for self-medication (Fisher’s exact test with Monte Carlo simulation, *p* < 0.001).

Finally, the occurrence of adverse reactions was significantly associated with subsequent consultation with an ophthalmologist (χ^2^(2) = 66.7, *p* < 0.001).

Due to incomplete responses to some questionnaire items, the sample size varied across analyses, as indicated in Table 3.

**Table 3:** Associations between demographic variables, perceptions, and self-medication-related outcomes.

Variables compared	Statistical test	χ^2^ (df)	*p*-value	*n*-analysed
Age × Self-medication	Pearson χ^2^	38.7 (5)	< 0.001	1,000
Gender × Self-medication	Pearson χ^2^	2.99 (1)	0.084	1,000
Education grouped × Self-medication	Pearson χ^2^	2.47 (1)	0.116	1,000
Safety perception × Adverse reactions	Pearson χ^2^	0.489 (2)	0.783	1,000
Problem type × Motivation	Fisher (Monte Carlo)	Not applicable	< 0.001	637
Adverse reactions × Consultation after use	Pearson χ^2^	66.7 (2)	< 0.001	688

Pearson’s chi-square test was applied when expected cell counts were adequate. Degrees of freedom are abbreviated as df (in parentheses). Fisher’s exact test with Monte Carlo simulation was used for contingency tables with multiple categories and low expected frequencies.

## Discussion

This study provides contemporary data on ophthalmic self-medication practices in Bulgaria and addresses an important evidence gap regarding self-directed use of eye medications in a European setting. The findings demonstrate a high prevalence of self-medication (63.7%), suggesting that the use of ophthalmic preparations without prior medical consultation is a common behavior in the surveyed population. In addition to prevalence, the study identified patterns related to symptoms, motivations, perceptions of safety, and subsequent healthcare-seeking behavior.

The observed prevalence is within the upper range reported in international studies of ophthalmic self-medication and exceeds estimates reported for general self-medication in several European populations (3, 8, 10). Differences between studies should be interpreted cautiously because prevalence estimates are influenced by study design, sampling methods, healthcare systems, regulatory environments, and the operational definition of self-medication used across studies (11–13). Nevertheless, the present findings indicate that ophthalmic self-medication is not restricted to low-resource settings and remains relevant in countries with formally structured healthcare systems.

Several factors may explain the frequent use of ophthalmic medications without prescription. The most commonly reported motivations in this study were lack of time to visit a specialist, perception that consultation was unnecessary, and difficulty obtaining an appointment. These findings suggest that both structural barriers and individual decision-making contribute to self-medication behavior. Similar drivers have been reported in other settings, where convenience, accessibility, cost, and perceived minor severity of symptoms influence treatment choices (4, 6, 8, 14).

Community pharmacists may play an important role within this context. Pharmacists are often the most accessible healthcare professionals and may represent the first point of contact for individuals with acute ocular symptoms. Appropriate pharmacist guidance can support safe self-care in minor conditions, promote correct product selection, and encourage timely referral when warning signs are present. At the same time, reliance on informal advice without adequate clinical assessment may delay diagnosis of more serious disorders. Previous Bulgarian data indicate that counseling practices in community pharmacies may vary, highlighting the importance of strengthening pharmacist training and interdisciplinary collaboration in this field (5, 15). Broader evidence also supports the relevance of pharmacists in guiding responsible self-medication and referral pathways (13).

Although most respondents considered self-medication safe, this perception should be interpreted carefully. Although reported adverse reactions were rare, this finding should be interpreted with caution. The low rate of self-reported complications may reflect underreporting, recall bias, or lack of clinical verification rather than true safety of self-medication practices. Many ocular conditions share similar symptoms despite different underlying causes, and empirical treatment based only on redness, irritation, or discomfort may not always be appropriate (2, 16, 17). The discrepancy between perceived safety and potential clinical risk highlights the need for targeted patient education.

The study also found that age was associated with self-medication behavior, whereas gender and education were not significant predictors in the grouped analyses. This may reflect differences in healthcare utilization, symptom interpretation, time constraints, prior treatment experience, or digital health information seeking across age groups (8, 9). These associations warrant further investigation in larger and more representative samples.

From a public health perspective, the findings support several practical recommendations. Public education initiatives should emphasize that persistent pain, photophobia, vision loss, trauma, purulent discharge, recurrent symptoms, or unilateral severe redness require prompt professional assessment. Clear guidance should also be provided regarding the limitations of over-the-counter ophthalmic products and the risks of using leftover or previously prescribed medications without re-evaluation. Community pharmacists should be supported with practical referral algorithms for red-flag symptoms, and efforts to improve timely access to ophthalmic care may reduce avoidable self-treatment (5, 13, 18).

This study has several strengths, including a large sample size and the simultaneous evaluation of behavioral, perceptual, and outcome-related aspects of ophthalmic self-medication within the same survey. However, important limitations should be acknowledged. The use of a convenience sampling strategy represents an important limitation, as the study population was predominantly female, highly educated, and digitally active, which may limit the generalizability of the findings to the broader Bulgarian population. In addition, all data were self-reported and may be affected by recall bias or social desirability bias. Clinical diagnoses, medication exposure, and adverse reactions could not be independently verified. The questionnaire was based on literature and internal review but was not externally validated. Finally, the cross-sectional design does not permit causal inference.

## Conclusion

Ophthalmic self-medication was highly prevalent in this Bulgarian sample, affecting nearly two-thirds of respondents. The practice was primarily driven by limited time for specialist consultation, perceived lack of necessity for medical evaluation, and barriers to timely access to ophthalmic care. Age was significantly associated with self-medication behavior, whereas gender and education were not.

Although many episodes may involve mild or self-limited symptoms, the high proportion of participants who perceived self-medication as safe suggests an important gap between confidence and potential clinical risk. Although adverse events were rarely reported, the discrepancy between perceived safety and self-assessed knowledge, together with the possibility of underreporting, suggests that the risks of ophthalmic self-medication may be underestimated. These findings highlight the need for improved public awareness regarding appropriate use of ophthalmic medications and recognition of warning symptoms requiring prompt professional assessment.

Future strategies should include targeted patient education, pharmacist-supported referral pathways, and improved access to timely eye care services in order to promote safer self-care practices and reduce avoidable delays in diagnosis and treatment.

## Data Availability

The raw data supporting the conclusions of this article will be made available by the authors, without undue reservation.
