# Supplementary material for: Patterns and prevalence of ophthalmic self-medication in Bulgaria: results from a cross-sectional survey
Source: Front Med (Lausanne). 2026 May 29;13:1830788. doi: 10.3389/fmed.2026.1830788 (PMC13259647; doi:10.3389/fmed.2026.1830788)
Supplement: Supplementary file 1 [file Table_1.DOCX]

**Selfmedication survey**

**Age**

**• Under 18**

**• 18-30**

**• 31-40**

**• 41-50**

**• 51-60**

**• 61-70**

**• Over 71**

**Biological sex**

**• Male**

**• Female**

**• Other**

**Education**

**• Primary education**

**• Secondary education**

**• Secondary specialized education**

**• Higher education**

**• Other:**

**Are you …?**

**• Student**

**• Working**

**• Unemployed**

**• Retired**

**• Other:**

**Have you used eye drops (eye drops) without a doctor's prescription?**

**• Yes**

**• No**

**If the answer to the previous question is "Yes", from whom did you receive a recommendation for the drops?**

**• Pharmacist**

**• Friends/relatives**

**• Internet**

**• Other:**

**What was the problem? What were the problems? (describe)**

**What is the main reason for treating yourself with eye drops that are not prescribed by a specialist?**

**• I did not have time to see a specialist**

**• I was unable to make an appointment with a specialist ophthalmologist**

**• I did not have time to make an appointment with a specialist ophthalmologist**

**• I did not consider it necessary to make an appointment with a specialist ophthalmologist**

**• The pharmacy knows very well what medicine to recommend to me**

**• I went to many eye doctors, but no one solved my problem**

**• I looked for a solution to the problem on the Internet**

**• Other:**

**What eye drops did you use without a doctor's prescription?**

**(You can choose more than one answer)**

**• Nonsteroidal anti-inflammatory drops (dicloabac, yelox, apfecto, medrolgin, ketorolac, uniclofen)**

**• Antibiotic drops (tobrex, tobrin, vigamox, oftaquix, levoximed, levofloxacin, floxal, isox, netacin, moxifloxacin, moxifloxacin, ofloxacin, ciloxan, ciprofloxacin, aziter)**

**• Combined antibiotic drops with corticosteroid (tobradex, tobodexin, dex-tobrin, ducresa, maxitrol, neladex, netildex, gentazone, hydrocortisone with chloramphenicol, spersadex, triovision)**

**• Anesthetics (alkaine, benoxy)**

**• Pure steroids (flarex, maxidex, softacort, dexanova, medexol, dexafry)**

**• Antiallergic (opatanol, zabac, olovision**

**• Glaucoma treatment drops (monoprost, xalatan, travatan, lumigan, latanoprost, timolol, oftan timolol, betoptik, azopt, cosopt, bimifry, amiptyfree, daveris-T, brytil, rimonal, brimogen,**

**• Vizin**

**• Artificial tears**

**• Others:**

**How often have you used eye drops without being prescribed by a doctor?**

**• Once**

**• Several times**

**• Often**

**• Never**

**• Other**

**How do you rate your own knowledge about the use of eye drops?**

**• Excellent**

**• Very good**

**• Good**

**• Average**

**• Bad**

**• Very bad**

**Do you think self-medication with eye drops is safe?**

**• Yes**

**• No**

**• I'm not sure**

**What is your opinion on the need for eye drops to be available with a prescription?**

**• Always needed**

**• Most of the time cases**

**• Not needed**

**• Not sure**

**Have you ever had any side effects or complications from using eye drops?**

**• Yes**

**• No**

**If yes, what are they?Анкета самолечение**

**Възраст**

- Под 18
- 18-30
- 31-40
- 41-50
- 51-60
- 61-70
- Над 71

**Биологичен пол**

- Мъжки пол
- Женски пол
- Друго

**Oбразование**

- Основно образование
- Средно образование
- Средно-специално образование
- Висше образование
- Друго:

**Вие сте …?**

- Студент
- Работещ
- Безработен
- Пенсионер
- Друго:

**Употребявали ли сте капки (колири) за очи без предписание от лекар?**

- Да
- Не

**Ако отговорът на предишния въпрос е "Да", от кого получихте препоръка за капките?**

- Фармацевт
- Приятели/роднини
- Интернет
- Друго:

**Какъв беше проблемът? Какви бяха проблемите? (опишете)**

**Каква е основната причина да се лекувате с капки за очи, които не са предписани от специалист?**

- Нямах време да ходя при специалист
- Не успях да запиша час при специалист офталмолог
- Нямах време да запиша час при специалист офталмолог
- Не смятах за необходимо за запиша час при специалист офталмолог
- От аптеката знаят добре какво лекарство да ми препоръчват
- Ходих при множество очни лекари, но никой не реши проблема ми
- Потърсих решение на проблема в интернет
- Друго:

**Какви капки за очи използвахте без лекарско предписание?**
(Имате възможност за повече от един отговор)

- Нестероидни противовъзпалителни капки (*диклоабак, йелокс, апфекто, медролгин, кеторолак, униклофен*)
- Антибиотични капки (*тобрекс, тобрин, вигамокс, офтакуикс, левоксимед, левофлоксан, флоксал, айсокс, нетацин, моксифлоксацин, моксифлоксан, офлоксацин, цилоксан, ципрофлоксацин, азитер)*
- Комбинирани капки антибиотик с кортикостероид *(тобрадекс, тободексин, декс-тобрин, дукреса, макситрол, неладекс, нетилдекс, гентазон, хидрокортизон с хлорамфеникол, сперсадекс, триовижън)*
- Упойки *(алкаин, бенокси)*
- Чисти стероиди *(фларекс, максидекс, софтакорт, дексанова, медексол, дексафри)*
- Противоалергични (опатанол, забак, оловижън
- Капки за лечение на глаукома ( монопрост, ксалатан, траватан, лумиган, латанопрост, тимолол, офтан тимолол, бетоптик, азопт, косопт, бимифри, амиптифри, даверис-Т, бритил, римонал, бримоген,
- Визин
- Изкуствени сълзи
- Други:

**Колко често сте използвали капки за очи без да бъдат предписани от лекар?**

- Веднъж
- Няколко пъти
- Често
- Никога
- Друго

**Как оценявате собствените си познания относно употребата на капки за очи?**

- Отлични
- Много добри
- Добри
- Средни
- Лоши
- Много лоши

**Смятате ли, че самолечението с капки за очи е безопасно?**

- Да
- Не
- Не съм сигурен/на

**Какво е вашето мнение за необходимостта капките за очи да се предлагат с рецепта по лекарско предписание?**

- Нужна е винаги
- Нужна е в повечето случаи
- Не е нужна
- Не съм сигурен/на

**Случвало ли се е да имате нежелани реакции или усложнения от употребата на капки за очи?**

- Да
- Не

**Ако да, какви?**
